# Epidemiology, characteristics and treatment of patients with relapsing remitting multiple sclerosis and incidence of high disease activity: Real world evidence based on German claims data

**DOI:** 10.1371/journal.pone.0231846

**Published:** 2020-05-01

**Authors:** Christoph Ohlmeier, Holger Gothe, Judith Haas, Ulrike Osowski, Carina Weinhold, Sarah Blauwitz, Niklas Schmedt, Wolfgang Galetzka, Fabian Berkemeier, Björn Tackenberg, Martin Stangel

**Affiliations:** 1 Department Health Services Research, IGES Institut GmbH, Berlin, Germany; 2 Chair for Health Sciences / Public Health, Medical Faculty “Carl Gustav Carus”, Technical University Dresden, Dresden, Germany; 3 Department of Public Health, Health Services Research and Health Technology Assessment, Institute of Public Health, Medical Decision Making and Health Technology Assessment, UMIT – University for Health Sciences, Medical Informatics and Technology, Hall i.T., Austria; 4 Center for Multiple Sclerosis, Jewish Hospital Berlin, Berlin, Germany; 5 Merck Serono GmbH, Darmstadt, Germany, an affiliate of Merck KGaA, Darmstadt, Germany; 6 InGef - Institute for Applied Health Research Berlin GmbH, Berlin, Germany; 7 Center of Neuroimmunology, Philipps-University, Marburg, Germany; 8 Clinical Neuroimmunology and Neurochemistry, Dept. of Neurology, Hannover Medical School, Hannover, Germany; University of Oxford, UNITED KINGDOM

## Abstract

**Background:**

Multiple Sclerosis (MS) is a chronic inflammatory, immune mediated disease of the central nervous system, with Relapsing Remitting MS (RRMS) being the most common type. Within the last years, the status of high disease activity (HDA) has become increasingly important for clinical decisions. Nevertheless, little is known about the incidence, the characteristics, and the current treatment of patients with RRMS and HDA in Germany. Therefore, this study aims to estimate the incidence of HDA in a German RRMS patient population, to characterize this population and to describe current drug treatment routines and further healthcare utilization of these patients.

**Methods:**

A claims data analyses has been conducted, using a sample of the InGef Research Database that comprises data of approximately four million insured persons from around 70 German statutory health insurances (SHI). The study was conducted in a retrospective cohort design, including the years 2012–2016. Identification of RRMS population based on ICD-10 code (ICD-10-GM: G35.1). For identification of HDA, criteria from other studies as well as expert opinions have been used. Information on incidence, characteristics and current treatment of patients with RRMS and HDA was considered.

**Results:**

The overall HDA incidence within the RRMS population was 8.5% for 2016. It was highest for the age group of 0–19 years (29.4% women, 33.3% men) and lowest for the age group of ≥ 50 years (4.3% women, 5.6% men). Mean age of patients with RRMS and incident HDA was 38.4 years (SD: 11.8) and women accounted for 67.8%.

Analyses of drug utilization showed that 82.4% received at least one disease-modifying drug (DMD) in 2016. A percentage of 49.8% of patients received drugs for relapse therapy. A share of 55% of RRMS patients with HDA had at least one hospitalization with a mean length of stay of 13.9 days (SD: 18.3 days) in 2016. The average number of outpatient physician contacts was 28.1 (SD: 14.0).

**Conclusions:**

This study based on representative Germany-wide claims data from the SHI showed a high incidence of HDA especially within the young RRMS population. Future research should consider HDA as an important criterion for the quality of care for MS patients.

## Introduction

Multiple Sclerosis (MS) is a chronic inflammatory, immune mediated disease of the central nervous system (CNS) and the most common cause of neurological disability in young adults [1]. MS is characterized by unpredictable episodes of CNS inflammation, so-called relapses, which lead to injuries of the myelin sheaths, oligodendrocytes, nerve cells and axons [1]. Clinical phenotypes can be categorized into four categories: the clinically isolated syndrome (CIS), relapsing-remitting MS (RRMS), secondary progressive MS (SPMS), and primary progressive MS (PPMS). RRMS is the most common disease type and often defines the initial disease phase for the majority of patients [2]. Without adequate treatment, more than half of all RRMS patients will develop a secondary progressive MS form [3].

MS affects approximately 2.3 million people worldwide, with large country specific disparities in prevalence [4]. Prevalence and incidence estimates for Germany vary depending on year, region and dataset, with more recent studies showing a prevalence of about 220,000 persons (0.32%) and an incidence rate of 18/100,000 persons for 2015 [5]. In 70% of cases, disease onset occurs between the age of 20–40 while women are affected 2.5 times more often than men [5,6]. Estimates of societal costs range between 28,000-63,000 € per patient depending on the disease-severity [7].

MS treatment aims to reduce the risk of relapses and disability progression. Within the last few years an increasing availability of disease modifying drugs (DMTs) has expanded treatment options towards a more individualized therapy. Recent recommendations for the assessment of the individual MS status not only include the disease course but also disease activity as a major component to determine the best treatment option [2,8]. Since newer drugs showed more favorable results e. g. regarding the reduction of relapses in the subgroup of RRMS patients with high disease activity (HDA) in clinical studies [9], HDA is now part of the definition of indication areas and is also used as a decision criterion for the selection of a specific drug or a group of drugs in current guidelines [8–10].

This implies a need for an improvement of the assessment and monitoring of the disease status and activity in addition to the classification of phenotypes [2,10,11].

RRMS patients with high disease activity (HDA) are characterized by a high relapse rate and an increasing progression of disability. Although HDA has become an increasingly important indicator for clinical decisions, little is known about the incidence, the characteristics, and the current treatment of patients with RRMS and HDA. To the best of our knowledge, there are no other studies describing the incidence of HDA within an RRMS population based on claims data.

The aim of this study is (i) to estimate the incidence of HDA in a German RRMS patient population, (ii) to characterize RRMS HDA patients regarding their demographic characteristics and diagnosed comorbidities and (iii) to describe current drug treatment routines and other healthcare utilization of these patients.

## Patients and methods

### Data source

Source of data was the InGef (Institute for Applied Health Research Berlin) Research Database. The InGef Research Database is an anonymized claims database comprising longitudinal data from approximately 6.7 million persons insured in one of the 70 German statutory health insurances (SHI) contributing data to the database. For the purpose of this analysis, the InGef Research Database was condensed to a sample of approximately 4 million insured people which is considered to be a representative of the German population in terms of age and sex and shows high external validity regarding overall measures of morbidity, mortality, and drug use as described elsewhere [12]. In brief, the InGef Research Database contains socio-demographic information such as age, sex, and the region of residence. Besides, the database gives information on hospitalizations, outpatient physician visits, and outpatient drug prescriptions. The hospital data comprises information on the date of admission and discharge, the reason for discharge, diagnostic and therapeutic procedures with the exact date as well as diagnoses which can be distinguished in hospital main discharge diagnoses and secondary diagnoses. The outpatient data also comprises information on diagnostic and therapeutic information with their exact date. Outpatient diagnoses can be distinguished into confirmed diagnoses, suspected diagnoses, status post diagnoses, and diagnoses ruled out. Inpatient and outpatient diagnoses are coded according to the German Modification of the International Classification of Diseases, 10^th^ Revision (ICD-10-GM). Data on outpatient prescription of reimbursed drugs comprise information on the date of prescription and dispensation as well as the pharmaceutical reference number. Based on a pharmaceutical reference database information on the anatomical-therapeutical-chemical code (ATC-code), the defined daily dose (DDD), the packaging size, as well as the strength and formulation of the drug can be linked for each prescribed drug. Furthermore, information on the specialty of care providing physicians can be obtained from the database [12].

Further clinical data, e.g. laboratory parameters, outcomes of diagnostic interventions, or relapses are not documented in this data source since this information has no relevance for the reimbursement of performed health services. At the time the study was conducted, data years 2012–2016 were available for analyses.

Data contributing to the InGef database are stored at a specialized data center according to §284 in combination with §70 and §71 Social Code Book (“Sozialgesetzbuch”, SGB) V. The data center is owned by SHIs and provides data warehouse services. In the data center (acting as a trust center), data with respect to individual insured members and health care providers (e.g. physicians, practices, hospitals, pharmacies) are anonymized by coarsening or by removing individual variables. Since all patient-level data in the InGef database are no longer social data according to § 67 Abs. 2 SGB X in combination with Art. 4 Nr. 1 of the General Data Protection Legislation (“Datenschutz-Grundverordnung”, DSGVO), institutional review board/ ethical approval and informed consent of the patient was not required.

### Study design

The study was conceived as a retrospective cohort design. Analyses were based on the years 2012-2016 while each year was considered as a separate observation period.

### Study population

Patients were included in the overall study population if they had continuous insurance coverage during the year preceding the observation period, during the actual observation period, or until death during the observation period, respectively. Furthermore, insurants had to have a documented diagnosis of multiple sclerosis (ICD-10-GM: G35.). To avoid misclassification all persons had to have at least one hospital main discharge diagnosis of MS or two diagnoses of MS (hospital secondary diagnosis or confirmed outpatient diagnosis) within one quarter or in two subsequent quarters of a year. Furthermore, identified MS patients were checked with regard to the presence of relapsing remitting MS (RRMS). To be identified as having RRMS, patients had to have a documented diagnosis of RRMS (ICD-10-GM: G35.1) and no diagnoses of secondary progressive MS (SPMS) (ICD-10-GM: G35.3). In case that a patient had documented diagnoses of RRMS and primary progressive MS (PPMS) (ICD-10-GM: G35.2) during the observation period, an MS patient was assigned to RRMS if RRMS was more frequently documented as hospital main discharge diagnosis or -if RRMS and PPMS were coded with the same frequency- if RRMS was the most current documented diagnosis. Assignment to RRMS was evaluated for each observation period separately. In addition, the study population was reduced to individuals with RRMS and incident HDA. For this purpose, the patients were not allowed to fulfill the HDA criteria (HDA criteria see below) prior to the first day of HDA in the observation period.

### Identification of high disease activity (HDA)

A commonly accepted definition of HDA does currently not exist. However, based on definitions used in clinical MS studies [13,14] and workshops with clinical experts in the field of MS treatment, this study identified RRMS patients suffering from HDA if one of the following criteria was met:

- Two relapses during a period of twelve months. The observation period was checked for relapses (definition of relapse see below). In case of an identified relapse, the presence of at least another relapse in the twelve months preceding this relapse was checked. The date of the second relapse was defined as the beginning of HDA.
- At least one relapse under treatment with a disease-modifying drug (DMD) during the observation period (definition of treatment duration see below). The date of relapse under DMD treatment was defined as the beginning of HDA.
- Prescription of alemtuzumab (ATC code: L01XC04), fingolimod (ATC code: L04AA27), which is only approved as secondary therapy in Germany, or natalizumab (ATC code: L04AA23), since guidelines recommend these drugs for the treatment of MS patients with HDA only [8–10]. The date of the respective first prescription was defined as the beginning of HDA.

Patients defined as having HDA in a specific observation period were classified as also having HDA in the following observation periods and were thus only identified as having initial signs of (incident) HDA in the first observation period in which one of the HDA criteria were met.

### Identification of relapses

In the currently used ICD-10-GM catalogue the 5^th^ digit of the ICD code refers to an acute exacerbation or progression. However, preliminary analysis suggested that relapses were not coded reliably this way. Therefore, we made use of surrogate parameters described in the literature for identification of relapses [15,16]. A relapse was assumed if at least one of the following criteria was met:

- Hospitalization due to MS (MS as main discharge diagnosis). If a urinary tract infection, cystitis, or pneumonia was documented as a secondary diagnosis, a pseudo-relapse was assumed and thus not counted as a relapse.
- Hospitalization associated with MS (MS as secondary diagnosis) and high-dose corticosteroid therapy during the hospital stay (identified via procedural codes) or during the first seven days after hospital discharge (identified via outpatient drug prescriptions).
- Confirmed outpatient diagnosis of MS and high-dose corticosteroid therapy (identified via outpatient drug prescriptions) within the quarter of the diagnosis.

High-dose corticosteroid therapy had to exceed at least 1,500 mg within 10 days after first prescription (methylprednisolone equivalent), since guidelines recommended this amount as the lower limit for corticosteroid therapy [8]. Analogous to previous studies, two identified relapses within a range of 30 days were considered as one relapse [15,16].

### Modeling of treatment duration

To calculate the range of a DMD prescription, the packaging size was multiplied by the number of DDD of one tablet or unit of other modes of application, respectively. A continuous DMD therapy was assumed if a further DMD prescription during the treatment duration of the previous prescription or within a period of no longer than 60 days (“Grace Period”) after the end of the previous prescription could be identified. Days of overlapping treatment durations of two prescriptions were added to the treatment duration. According to label treatment, duration with alemtuzumab should be 12 months. In case of a further treatment with alemtuzumab between the 10^th^ and 14^th^ month after treatment initiation a continuous alemtuzumab therapy up to the end of the observation period was assumed if no other DMD was prescribed afterwards. In case of a prescription of other DMD than alemtuzumab, discontinuation of alemtuzumab treatment was assumed.

Apart from alemtuzumab, fingolimod, and natalizumab, which can also be identified in the inpatient setting, only outpatient prescriptions were considered to calculate the treatment duration.

### Patient characteristics

Characteristics of RRMS patients with incident HDA were assessed based on data of the most current observation period. Hospital main or second discharge diagnoses and confirmed outpatient diagnoses were considered to describe the frequency of pre-specified diseases and to analyze the general comorbidity (via Elixhauser comorbidity score, ECS) [17] in RRMS patients with incident HDA. Information on DMD treatment, corticosteroid treatment and pre-specified comedication were identified based on outpatient prescribed drugs and, if separately coded, inpatient dispensed drugs. Documented other care services (medical aids, remedies) were also used to characterize RRMS patients with incident HDA. Comorbidities, use of drugs and use of other care services were not defined as exclusive groups. Thus, one patient could have received more than one drug and could have had more than one comorbidity.

### Statistical analyses

The incidence of HDA in 2016 within the group of RRMS patients was calculated by dividing the number of patients with incident HDA by the number of RRMS patients stratified by sex and age. The incidence rate was given in percent. Corresponding 95% confidence intervals (CIs) were calculated according to the substitution method [18]. The prevalence of specific comorbidities, the use of drugs and other health care services was calculated by dividing the number of patients fulfilling the criteria of the respective indicator by the total number of RRMS patients with incident HDA. To characterize the intensity of health care utilization, the mean and the standard deviation (SD) was calculated. Statistical tests regarding e. g. differences between groups were not performed, since the analyses were purely explorative. Statements regarding the statistical significance of observed differences can therefore not be made.

## Results

### Patient characteristics

We identified 500 patients with RRMS and initial signs of HDA (= incident) for the year 2016 within our overall study sample. Of those, 27.4% were identified as having HDA due to at least two observed relapses (Table 1). At least one relapse under treatment with DMD treatment led to case identification in 23.4% of the HDA patients. HDA identification due to a combination of criteria including at least one of the aforementioned criteria accounted for 42.0%. Only 7.2% of HDA were only identified due to the receipt of escalation drugs.

**Table 1:** Quantification of criteria leading to identification of HDA in 2016.

	All (n = 500)	
	n	%
Criteria for HDA identification		
At least two relapses	137	27.4%
At least one relapse under treatment with DMD	117	23.4%
Receipt of escalation therapy	36	7.2%
Combination of at least two relapses and at least one relapse under treatment with DMD	141	28.2%
Other combinations of criteria	69	13.8%

DMD: Disease-modifying drug

Women accounted for 67.8% of the RRMS HDA population. Mean age was 38.4 years (SD: 11.8), showing no differences between female and male patients (Table 2).

**Table 2:** Demographics of patients with RRMS and incident HDA in 2016.

	Women (n = 339)		Men (n = 161)		All (n = 500)	
	n	%	n	%	n	%
Age						
0–19 years*	15	4.4%	5	3.1%	20	4.0%
20–29 years	70	20.6%	40	24.8%	110	22.0%
30–39 years	102	30.1%	42	26.1%	144	28.8%
40–49 years	91	26.8%	40	24.8%	131	26.2%
≥50 years**	61	18.0%	34	21.1%	95	19.0%
All	339	100%	161	100%	500	100%
Mean age (mean, SD)	38.4 (+/-11.8)		38.4 (+/-11.7)		38.4 (+/-11.8)	
Sex						
Female					339	67.8%
Male					161	32.2%

*Of those aged 0–19 years 15 patients were aged <18 years.

**Numbers and proportions of other age groups cannot be displayed due to data protection reasons.

RRMS: relapsing remitting multiple sclerosis; HDA: high disease activity; SD: Standard deviation.

The mean number of other diagnosed comorbidities of the ECS alongside MS was 2.9 (SD: 1.7) for women and 2.6 (SD: 1.6) for men. All HDA RRMS patients had been diagnosed for at least one other disease of the ECS, while the number of comorbidities was between 1–3 for most of the RRMS patients (Table 3). Most frequently diagnosed specific comorbidities were depression (32.2%) and anxiety (15.8%) as well as hypertension (17.8%). While depression and anxiety had been diagnosed more often in women than in men (36.9% and 22.4% in women; 17.7% and 11.8% in men, respectively), diagnosed hypertension was identified slightly more often in men than in women (19.9% and 17.1%, respectively).

**Table 3:** Comorbidity of patients with RRMS and incident HDA in 2016.

	Women (n = 339)		Men (n = 161)		All (n = 500)	
	n	%	n	%	n	%
Elixhauser comorbidity score						
0 diseases	0	0.0%	0	0.0%	0	0.0%
1 disease	74	21.8%	43	26.7%	117	23.4%
2 diseases	96	28.3%	49	30.4%	145	29.0%
3 diseases	75	22.1%	37	23.0%	112	22.4%
4 diseases	34	10.0%	11	6.8%	45	9.0%
5 diseases	31	9.1%	10	6.2%	41	8.2%
6+ diseases	29	8.6%	11	6.8%	40	8.0%
Mean number of diseases (mean, SD)	2.9 (+/-1.7)		2.6 (+/-1.6)		2.8 (+/-1.7)	
Specific comorbidity						
Anxiety	60	17.7%	19	11.8%	79	15.8%
Asthma	34	10.0%	14	8.7%	48	9.6%
Cancer	15	4.4%	8	5.0%	23	4.6%
Cystitis	*	*	*	*	29	5.8%
Depression	125	36.9%	36	22.4%	161	32.2%
Diabetes mellitus	15	4.4%	9	5.6%	24	4.8%
Hashimoto's disease	*	*	*	*	22	4.4%
Heart rhythm disorders	*	*	*	*	20	4.0%
Hypertension	58	17.1%	31	19.3%	89	17.8%
Liver diseases	14	4.1%	9	5.6%	23	4.6%
Psoriasis	*	*	8	5.0%	16	3.2%
Urinary tract infection	*	*	*	*	44	8.8%

*Numbers and proportions cannot be displayed due to data protection reasons.

RRMS: relapsing remitting multiple sclerosis; HDA: high disease activity; SD: Standard deviation.

### Incidence of HDA in RRMS patients

Overall HDA incidence of included RRMS patients was 8.5% for 2016, while large disparities according to patients age could be identified (Table 4). Age specific incidence of HDA within the RRMS population was highest for the age group of 0–19 years with 33.3% for men and 29.4% for women. Incidence decreased with increasing age and was lowest for the group aged ≥ 50 years where 4.3% of the female and 5.6% of the male RRMS patients had incident HDA. In almost every age group HDA incidence was slightly higher for men than for women.

**Table 4:** Incidence of HDA in patients with RRMS in 2016 (n = 500).

	Women			Men			All		
	RRMS population (n)	Incident HDA (n)	%	RRMS population (n)	Incident HDA (n)	%	RRMS population (n)	Incident HDA (n)	%
0–19 years	51	15	29.4%	15	5	33.3%	66	20	30.3%
20–29 years	478	70	14.6%	213	40	18.8%	691	110	15.9%
30–39 years	968	102	10.5%	433	42	9.7%	1,401	144	10.3%
40–49 years	1,216	91	7.5%	531	40	7.5%	1,747	131	7.5%
≥50 years	1,406	61	4.3%	604	34	5.6%	2,010	95	4.7%
All	4,119	339	8.2%	1,796	161	9.0%	5,915	500	8.5%

RRMS: relapsing remitting multiple sclerosis; HDA: high disease activity

### Drug treatment and other healthcare utilization

The data show that 82.4% of RRMS patients with HDA received at least one disease-modifying drug in 2016 with men (85.1%) receiving DMD treatment slightly more often than women (81.1%) (Table 5). First-line DMD therapy was observed in 69.8% of all RRMS patients with incident HDA. Interferon beta was the most frequently observed first-line therapy (26.4%). Glatiramer acetate and dimethyl fumarate were used in 22.6% and 15.8% of RRMS patients with incident HDA, respectively. Sex-specific differences regarding the use of first-line therapy drugs were rarely observed. Use of dimethyl fumarate was higher in women than in men (17.4% and 12.4%, respectively), whereas men more often received teriflunomide than women (14.9% and 8.0%, respectively).

**Table 5:** Drug treatment of patients with RRMS and incident HDA in 2016.

	Women (n = 339)		Men (n = 161)		All (n = 500)	
	n	%	n	%	n	%
Overall DMD therapy **						
Any DMD	275	81.1%	137	85.1%	412	82.4%
Any first-line therapy	236	69.6%	113	70.2%	349	69.8%
Any second-line therapy	66	19.5%	39	24.2%	105	21.0%
First-line treatment **						
dimethyl fumarate	59	17.4%	20	12.4%	79	15.8%
glatiramer acetate	75	22.1%	38	23.6%	113	22.6%
interferon beta	95	28.0%	37	23.0%	132	26.4%
Second-line treatment **						
alemtuzumab	*	*	*	*	6	1.2%
fingolimod	42	12.4%	28	17.4%	70	14.0%
natalizumab	23	6.8%	10	6.2%	33	6.6%
Relapse therapy **						
Any relapse therapy	175	51.6%	74	46.0%	249	49.8%
methylprednisolone	114	33.6%	45	28.0%	159	31.8%
prednisolone	68	20.1%	29	18.0%	97	19.4%
Immunoadsorption/Plasma exchange	10	2.9%	6	3.7%	16	3.2%

*Numbers and proportions cannot be displayed due to data protection reasons.

**One patient may have received more than one drug / therapy.

RRMS: relapsing remitting multiple sclerosis; HDA: high disease activity; DMD: Disease-Modifying Drug

Second-line therapy was received by 21.0% of all RRMS patients with incident HDA with a more frequent use observed in men compared to women (24.2% and 19.5%, respectively). Fingolimod was the most frequently observed second-line therapy (14.0%).

A percentage of 49.8% of patients received drugs for relapse therapy with a more frequent use in women compared to men (51.6% and 46.0%, respectively). Methylprednisolone was the most frequently observed corticosteroid (31.1%).

Concerning health service utilization it was observed that 55% of the HDA RRMS patients had at least one hospitalization in 2016 with a mean length of stay of 13.9 days (SD: 18.3 days) (Table 6). Hospitalizations due to MS were seen in 42.2% of the patients. With view to outpatient health service utilization, the average number of physician contacts was 28.1 (SD: 14.0), while the number was slightly higher for female than male patients (29.9 vs. 24.1, respectively). Furthermore, a percentage of 69.3% of female and 64.6% of male persons had at least one contact with an outpatient neurologist. On average the neurologist was consulted 8.5 times (SD: 6.1) by RRMS patients with incident HDA within 2016. Regarding procedures most patients (84.8%) had at least one MRI, while patients had on average three (2.9, SD: 1.9) MRIs within one year. Lumbar puncture was done for 25.8% of patients with a more frequent use in men than in women (32.3% vs. 22.7%, respectively).

**Table 6:** Other health service utilization of patients with RRMS and incident HDA in 2016.

	Women (n = 339)		Men (n = 161)		All (n = 500)	
	n	%	n	%	n	%
Hospitalizations						
At least one hospitalization	179	52.8%	96	59.6%	275	55.0%
Number of hospitalizations (mean, SD)	1.9 (+/-1.2)		1.8 (+/-1.1)		1.8 (+/-1.2)	
Length of stay (mean, SD)	14.4 (+/-20.1)		13.0 (+/-14.5)		13.9 (+/-18.3)	
Hospitalizations due to MS						
At least one hospitalization	137	40.4%	74	46.0%	211	42.2%
Number of hospitalizations (mean, SD)	1.5 (+/-0.8)		1.6 (+/-1.0)		1.6 (+/-0.9)	
Length of stay (mean, SD)	12.3 (+/-17.6)		10.7 (+/-9.5)		11.7 (+/-15.2)	
Outpatient care services						
At least one physician contact	339	100.0%	160*	99.4%	499	99.8%
Number of physician contacts (mean, SD)	29.9 (+/-14.8)		24.1 (+/-11.1)		28.1 (+/-14.0)	
At least one contact with neurologist	235	69.3%	104	64.6%	339	67.8%
Number of neurologist contacts (mean, SD)	8.1 (+/-6.1)		9.5 (+/-5.9)		8.5 (+/-6.1)	
Procedures						
At least one MRI	290	85.5%	134	83.2%	424	84.8%
Number of MRIs (mean, SD)	2.8 (+/-1.8)		3.0 (+/-2.0)		2.9 (+/-1.9)	
At least one lumbar puncture	77	22.7%	52	32.3%	129	25.8%
Number of lumbar punctures (mean, SD)	1.1 (+/-0.4)		1.1 (+/-0.3)		1.1 (+/-0.3)	

*Absence of outpatient physician contact in n = 1 male patient might have occurred due to e. g. a short observation period due do change of health insurance company / death or only inpatient therapy.

RRMS: relapsing remitting multiple sclerosis; HDA: high disease activity; SD: Standard deviation.

## Discussion

Based on data from a large German health insurance database we analyzed drug use and health care utilization of RRMS patients with HDA and also estimated the incidence of HDA within the RRMS population. We identified 8.5% of RRMS patients as being HDA incident within the year 2016. Most of HDA incident RRMS patients (82.4%) received DMDs with interferon beta as the most frequently used drug for first-line therapy and fingolimod as the most frequently used drug for second-line therapy. Analysis of health care utilization showed that 42% of RRMS HDA incident patients had at least one hospitalization due to MS in 2016 and 68% of RRMS HDA incident patients had at least one outpatient neurological contact.

Characterization of HDA incident RRMS patients showed a higher percentage of women (67.8%) and a mean age of 38.5 years. Thus, patient age was slightly lower than reported for general RRMS populations based on a nationwide epidemiological German MS registry (42.7 years) that included patient data from more than 150 MS centers [19,20]. A lower mean age could be expected upfront, since we studied HDA incident cases. Higher percentages of women have also been described for general RRMS populations (73.1%) within registry studies [19,20].

We found an overall HDA incidence within the RRMS population of 8.5% for the year 2016, showing a strong decline with increasing age. While HDA incidence within the youngest age group of 0-19-year-old patients was 30.3%, patients aged ≥ 50 years showed an HDA incidence of 4.7% only. These results are plausible regarding our definition of HDA. We defined relapse frequency as one possible indicator for HDA incidence. Considering that relapse frequency is both, time and age dependent, with higher relapse rates in early stages of the disease course and a decline with increasing age [21], these results are not surprising. In addition, we defined the prescription of either alemtuzumab, fingolimod, or natalizumab or having one relapse under treatment of DMD as further indicators for HDA. Given the fact that DMDs are typically given early in the disease course [8,10,22] higher incidence measurements in younger ages seem plausible.

Our study showed a large proportion of HDA incident RRMS patients (82.4%) receiving DMD therapy. A former study, also analyzing drug use based on claims data, showed a percentage of 58.6% of all RRMS patients receiving DMDs in 2009 [23]. Our findings thus indicate a higher DMD use amongst HDA RRMS patients. Since claims data analysis showed an increasing DMD use over time [23], availability of DMDs has increased over the last years and since our study focused on the subpopulation HDA patients, a higher proportion of DMD treated patients in our study was to be expected. Comparing our findings to the results of registry studies, our proportion of 82.4% of HDA incident RRMS patients receiving DMDs are in line with the reported proportion of 85.3% (95%-CI: 84.1–86.5%) of all RRMS patients receiving DMD-treatment [24]. Nevertheless, registry data and SHI database may be not directly comparable due to divergent selection of patient populations. While claims databases comprise all persons insured in specific health insurance companies, registries are likely to include patients from specialized centers who provide a quality of care, which is likely to differ from routine care outside of specialized centers and might also attract patients with a comparatively severe course of disease.

Focusing on the use of specific drugs in HDA incident RRMS patients, previously conducted claims data [23,25,26] or registry data analysis [6,24] did not specifically focus on RRMS but on overall MS populations. Therefore, results are not directly comparable. Furthermore, earlier analyses may not include DMDs that became available more recently. Comparing our results to more recently published studies based on the German National MS Cohort, a multicenter prospective cohort study [27], our findings on use of DMDs for first-line therapy are in agreement. The authors reported the use of dimethyl fumarate in 12.8% (15.8% in our study), glatiramer acetate in 22.4% (22.6% in our study), and interferon beta in 52.7% (26.4% in our study) of RRMS patients. Results for DMDs used for second-line therapy differ though, which is most likely because Bismarck et al. focus on initial DMD treatment. Nevertheless, results on the use of DMDs for second-line therapy within general MS populations based on a European wide MS survey [7] are very close to those that are reported in our study. For second-line therapy within our HDA RRMS population, fingolimod was prescribed more often than natalizumab (14% vs. 6.6% respectively) while Flachenecker et al. described fingolimod use in 12.6% and natalizumab use in 7.9% of MS patients [7]. The fact that a higher proportion of use of specific drugs for second-line therapy in the HDA population could not be shown compared to the general MS population may be due to the low response rate and the possibly associated selection of the sample, e. g. due to a possibly higher probability of participation of patients treated by neurological specialists.

Furthermore, almost half of the HDA RRMS patients (49.8%) within our study received relapse therapy, while Methylprednisolone was the most commonly prescribed drug (31.8%). Compared to calculations for a general MS population, where 23.4% of the patients received relapse therapy [26], our number is higher. This could be expected upfront as we were looking at HDA RRMS patients being defined amongst other criterions by a more frequent occurrence of relapses.

About half of the patients considered as having HDA incident RRMS status within this study had at least one hospitalization in 2016 and a relatively high number of physician contacts (28.1, SD: 14.0). These numbers are not surprising since recent studies on resource utilization and costs of MS also have shown that Germany has high hospitalization and consultation rates for MS patients in general [7]. Also, as MS patients with HDA are likely to have a more intensive resource utilization, our results seem to be plausible.

Interestingly, only 69.3% of female and 64.6% of male patients within the HDA RRMS population had contact with a neurologist within 2016. These proportions seem quite low with regards to neurologists as being specialized physicians for diagnosis, treatment, and monitoring of MS. However, since we were only analyzing outpatient services, patients might also have had neurologist consultations in an inpatient care setting that has not been assessed within our study, since information on the specialty of the treating physicians in hospital or the specialization of the ward was not comprised by our database. When looking at MRIs, conducted for RRMS patients with incident HDA in 2016, it becomes apparent that most patients had this kind of procedure (84.8%). Given the fact that recent recommendations advocate monitoring disease activity within DMD treated patients by both, clinical and MRI assessment [10], the number seems to be plausible.

Given the fact that there is currently no consensus definition of high active RRMS, numbers strongly depend on the definition used for HDA [28]. In this study, a validated definition based on clinical MS studies and expert opinions was used to identify HDA incident cases within the RRMS population [15,16]. Nevertheless, numbers might deviate from studies in which clinical information (e. g. results from MRI) are available.

We used a cross-sectional study design to analyze the routine treatment of RRMS patients with incident HDA with the calendar year as the observation period. It may therefore be that we identified drug dispensations that were prior to the first appearance of HDA.

Claims data such as the data source used for this study generally only comprise those information which are relevant for reimbursement purposes. Therefore, care services which are not covered by the SHI or comorbidities which cannot be documented according to ICD-10 codes or do not make a physician contact necessary can only be analyzed to a limited extent and might lead to an underestimation of respective results. However, as RRMS and particularly RRMS with HDA is a severe disease and disease-modifying treatment options are covered by the SHI, our results are unlikely to be affected by these general limitations of claims data.

## Conclusions

Our study, which was based on representative Germany-wide claims data from statutory health insurance funds, showed a high incidence of HDA within the RRMS population which decreased with age. Future studies on the quality of care for patients with MS should consider HDA as a criterion, as current guidelines make recommendations also based on HDA status.
